# Effects of High Affinity Leptin Antagonist on Prolactin Receptor Deficient Male Mouse

**DOI:** 10.1371/journal.pone.0091422

**Published:** 2014-03-25

**Authors:** Nadège Carré, Gili Solomon, Arieh Gertler, Nadine Binart

**Affiliations:** 1 Inserm U693, Le Kremlin-Bicêtre, France; 2 Université Paris-Sud, Faculté de Médecine Paris-Sud, UMR-S693, Le Kremlin-Bicêtre, France; 3 The Hebrew University of Jerusalem, Institute of Biochemistry, Food Science and Nutrition, Rehovot, Israel; 4 Assistance Publique-Hôpitaux de Paris, Hôpital de Bicêtre, Service d’Endocrinologie et des Maladies de la Reproduction, Le Kremlin Bicêtre, France; Hosptial Infantil Universitario Niño Jesús, CIBEROBN, Spain

## Abstract

Hyperprolactinemia occurs during gestation and lactation with marked hyperphagia associated with leptin resistance. Prolactin (PRL) induces the expression of orexigenic neuropeptide Y (NPY) in hypothalamic dorsomedial nucleus (DMH) leading to hyperphagia. Along this line prolactin receptor deficient (PRLR^−/−^) mice are resistant to obesity under high fat diet due to increased energy expenditure. As these mice have an altered food intake, our objective was to test whether leptin is responsible for these characteristics. PRLR^−/−^ male mice and control littermates were injected subcutaneously every other day with 12 mg/kg pegylated superactive mouse leptin antagonist (PEG-SMLA) for 3 weeks. We tested the effect of PEG-SMLA on body weight, food intake and metabolic parameters. The antagonist led to a rapid increase in body weight (20%) but increased adipose mass in PEG-SMLA treated mice was less pronounced in PRLR^−/−^ than in WT mice. Food intake of PEG-SMLA-injected animals increased during the first week period of the experiment but then declined to a similar level of the control animals during the second week. Interestingly, PRLR^−/−^ mice were found to have the same bone volume than those of control mice although PEG-SMLA increased bone mass by 7% in both strains. In addition, PEG-SMLA led to insulin resistance and glucose intolerance as well as an altered lipid profile in treated mice. Altogether, these results suggest that PRLR^−/−^ mice respond to leptin antagonist similarly to the control mice, indicating no interaction between the actions of the two hormones.

## Introduction

PRL is a hormone principally produced by the pituitary lactotroph cells. It acts by binding to a membrane receptor (PRLR) which is ubiquitously expressed conferring to PRL a large potential of action. Indeed, roles of PRL are multiple and are divided into several categories: reproduction, osmoregulation, immunoregulation, growth and development and metabolism [1], [2]. PRL is well known to induce metabolic changes notably during pregnancy and lactation where the supply for litters should be ensured. Furthermore, intracerebroventricular PRL administration induces enhanced food intake in rats, this hyperphagic effect is mediated by a PRL action on paraventricular nucleus [3]. PRL may have a direct effect on food intake through neuropeptideY expression in neurons coexpressing PRLR mRNA [4]. In addition, PRL may also impact energy homeostasis through modulation of lipid metabolism [5]. It was also demonstrated that hyperprolactinemia induces an insulin-resistant state in humans [6]. Moreover, patients with hyperprolactinemia have been described to exhibit altered energy metabolism and are candidates to obesity [7]. Treatment of these patients to normalize their PRL levels is accompanied by a reduction of body weight [8] and an improvement of glucose tolerance and insulin sensitivity. Indeed, cellular as well as transgenic animal models provided evidence that PRLR signaling exerts crucial roles in the development and function of two major players of whole body energy balance i.e. adipose tissues and endocrine pancreas. Two recent studies showed that rs4712652 SNP near the PRL gene showed association with BMI and risk of obesity [9], [10].

Recently, we showed that lack of PRLR causes resistance to high fat diet-induced obesity due to enhanced energy expenditure and increased metabolic rate. Mutant mice displayed reduced fat mass associated with appearance of massive brown-like adipocyte foci in fat depots under high fat diet [11]. Since these animals have an altered food intake our objective was to test whether leptin had an impact on their eating behavior. For this purpose, we used the recently developed mono-pegylated super active mouse leptin antagonist (D23L/L39A/D40A/F41A mutant of mouse leptin), termed PEG-SMLA [12] and treated the subgroups of both control and PRLR deficient mice to ameliorate the endogenous leptin signaling. We have recently shown that bone analyses revealed a significant increase in trabecular and cortical parameters measured in both the lumbar vertebrae and tibiae, in PEG-SMLA-treated mice in the first and third months, as well as a significant increase in tibia biomechanical parameters [13]. In this study we checked whether similar effect of PEG-SMLA occurs also in PRLR deficient mice.

## Materials and Methods

### Ethics Statement

In all experiments, animals were maintained under 12-h light/dark cycles, and were bred according to the Guide for the Care and Use of Laboratory Animals published by the US National Institute of Health (NIH Publication No. 85-23, revised 1996). The animal facility was granted approval (N°C94-043-12), given by the French Administration (Ministère de l’Agriculture). All procedures were approved by the local ethic committee Consortium des Animaleries Paris Sud (CAPSud) (N°2012-021).

### Animals and Treatment

Seven weeks-old male 129/SvJ mice wild type or PRLR^−/−^ were obtained in our local colony with free access to food and water. PEG-SMLA was prepared as described by Shpilman et al [12]. Mice were subcutaneously administered with PEG-SMLA at 12 mg/kg every other day for a period of 20 days. During this period, food intake and weight gain were recorded daily and averaged for a period of 7 days. Mice were killed after an intraperitoneal anesthesia (a ketamine/xylazine mix) and adipose tissue depots were weighted and frozen in dry ice.

### Isolation of Total mRNA and Analysis by qRT-PCR

Total RNA was extracted from adipose tissues using TRI Reagent solution (Life Technologies, Saint-Aubin, France) by homogenization with Tissuelyser (Qiagen, Courtaboeuf, France). Quantitative real-time PCR was performed as described previously [14]. After DNAse I treatment, RNA was reverse-transcribed and used for quantitative RT-PCR (qRT-PCR) using the Power SYBR® Green PCR Master Mix (Applied Biosystems, Courtaboeuf, France). Final primer concentrations were 300 nM (primer sequences are available upon request). Reaction parameters were carried out on a StepOne™ Real-Time PCR System (Applied Biosystems). Relative expression of *Leptin, Zfp423, PPARγ* and *aP2* genes within a given sample was calculated as a ratio (amol of specific gene/amol of 36B4). Results are presented as mean ± SEM.

### Histomorphological Procedures

Adipose tissues were fixed overnight in 4% paraformaldehyde in phosphate-buffered saline (PBS), dehydrated in 70% ethanol, and embedded in paraffin. Five-μm-thick sections were prepared and paraffin-embedded adipose sections were analyzed after hematoxylin and eosin staining. Six fields were randomly observed at a ×10 magnification of optical microscope (Provis, Olympus, Rungis, France). Adipocyte size quantification was performed using the ImageJ software (http://rsbweb.nih.gov/ij/) on at least >200 cells per mouse, leading to a mean adipocyte surface determination on 5000 to 8000 adipocytes per genotype.

### Analytical Procedures

Serum concentrations of triglycerides (TG), cholesterol (CH), non-esterified fatty acids (NEFA), alanine-aminotransferase (ALAT) and aspartate aminotransferase (ASAT) were determined using an automated Monarch device (Laboratoire de Biochimie, Faculté de Médecine Bichat, Paris, France). Serum insulin concentrations were determined using ultra-sensitive mouse insulin ELISA (Crystal Chem, Downers Grove, IL, USA) and mouse insulin ELISA (Mercodia, Uppsala, Sweden) using a mouse insulin standard, the assay range was 0.1–6.4 ng/mL and 0.2–6.5 ng/mL respectively.

### Oral Glucose Tolerance Tests (OGTT)

Glucose tolerance tests were performed 12 days after the beginning of the treatment by glucose gavage (2 g D-glucose/kg body weight) after an overnight fast. Blood glucose was determined using AccuCheck performa glucometer (Roche). Blood samples were collected before and at 30, 45, 60, 90 and 120 minutes after gavage.

### Homeostasic Model Assessment (HOMA) Determination

Insulin resistance was assessed by using the HOMA-IR as follows: fasting plasma glucose (mg/dl) × fasting insulin (mU/l)/405.

### Microcomputed Tomography (CT) Analysis of Trabecular Bones

Micro-CT analysis of third and fourth lumbar vertebrae (LV) was carried out as described recently [13]. Trabecular bone measurements in the lumbar vertebra included trabecular bone volume (BV)/tissue volume (TV) (%), trabecular number (Tb.N) (1/μm), trabecular thickness (Tb-Th) (μm), and trabecular separation (Tb.Sp) (μm).

### Statistical Analysis

Data are expressed as means ± SEM and analyzed using a two way ANOVA test with use of the computer software Prism 5 (GraphPad Software, San Diego, USA). Statistical significance is indicated at p values <0.05, 0.01 and 0.001.

## Results

### Leptin Antagonist Induces Dramatic Weight Gain in WT and PRLR^−/−^ Mice

In order to address the role of leptin in a PRL receptor deficient mouse model which is resistant to diet induced weight gain, we studied the effect of a PEG-SMLA on energy homeostasis in comparison with WT mice. The treatment effects on weight gain, food intake, adipose tissue morphology and development and energy homeostasis were determined.

**Figure 1:**
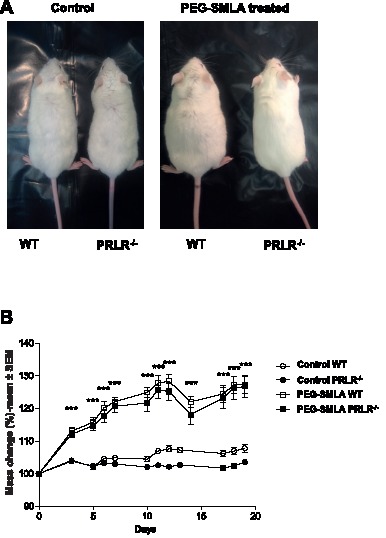
Effect of PEG-SMLA treatment on weight gain of WT and PRLR^−/−^ mice. (A) Representative dorsal view of male mice after 20 days of saline (control) or PEG-SMLA injections. (B) Comparison of PEG-SMLA effects on weight gain of WT (open squares) or PRLR^−/−^ (black squares) male mice. PEG-SMLA was injected daily at 6 mg/kg. ***, p<0.005 between non-treated (Cont) and PEG-SMLA treated groups.

**Figure 2:**
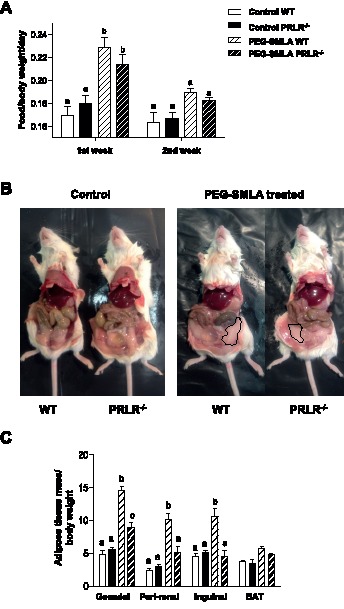
Effects of PEG-SMLA treatment on food intake and adipose tissues mass. (A) Food consumption (g) is calculated reported to body weight of each mouse on first or second week of treatment. PEG-SMLA injections are represented within hatched bars. b, p<0.001 related to Control WT mice. (B) At the end of treatment, the intact abdominal fat depot (delimited lines) is shown on both genotypes. (C) Ratio of adipose depot mass per body weight. b, p<0.001 and c, p<0.01 related to Control WT mice. (n = 7–10/group).

PEG-SMLA treatment induces a rapid and dramatic weight gain of almost the same order (20%) in WT and PRLR^−/−^ mice as shown in Figures 1A and 1B. PRLR^−/−^ mice exhibited a basal body weight lower than those of wild type mice [15], but PEG-SMLA treatment induced a similar highly significant (*p<*0.005) increased mass change in the WT and PRLR^−/−^ treated mice (Figure 1A), which was quantified in Figure 1B. During the first week of treatment, body mass change of injected mice increased about 20% while that of control mice did not change more than 3% (Figure 1B). These results paralleled the increased food intake. PEG-SMLA-treated mice, whatever the presence of PRLR, presented a drastic statistically significant increase of their food intake compared to saline injected mice during the first week of treatment (Figure 2A). The body weight gain and the food intake of the PEG-SMLA-treated mice during the second week were lesser than those of non-treated mice (Figure 2A). It is important to note that PEG-SMLA treatment exhibits reversible effects, and when treatment was stopped the body weight of formerly PEG-SMLA treated mice declined down to the values of control mice within 10 days (data not shown). After 20 days of treatment, dissection revealed that the increased adipose mass in PEG-SMLA treated mice was less pronounced in PRLR^−/−^ than in WT mice (Figure 2B).

We analyzed the interscapular Brown Adipose Tissue (BAT) and three major white fat depots: the peri-gonadal, peri-renal and inguinal adipose tissues.

Inguinal WAT is often defined as the depot attached dorsally along the pelvis and is routinely considered to represent subcutaneous adipose tissue in mice; it is the largest subcutaneous depot. Gonadal (epididymal) WAT is the largest visceral depot in male mice and embeds the vas deferens, testicular arteries and the epididymis. Perirenal WAT is a depot embedding the major part of the renal hilum of the kidney, where the renal artery enters and the ureter and renal vein exit the kidney. In WT mice, PEG-SMLA treatment induced an important increase of all white adipose tissues (WAT) with no effect on BAT (Figure 2C). However, PRLR^−/−^ treated mice showed smaller white fat pads than those of WT PEG-SMLA treated animals (Figure 2C). This indicates that PRLR^−/−^ mice responded to the leptin antagonist by increasing food intake with only a significant increase of the gonadal fat depot without changes in other fat depots.

### Analysis of Adipose Markers Gene Expression and Adipocyte Morphology

**Figure 3:**
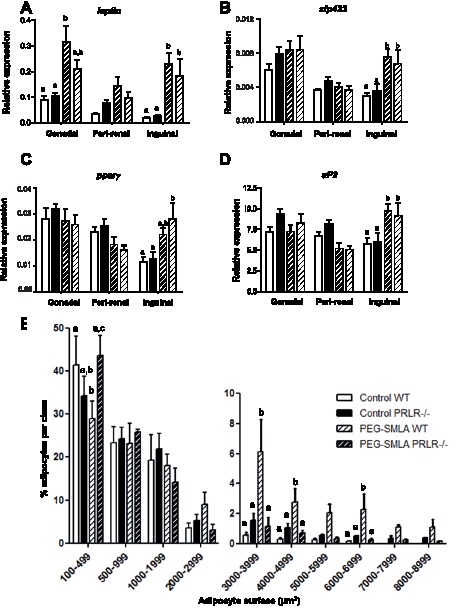
Expression of key adipose genes. (A–D) *Leptin, Zfp423, PPAR* and *aP2* gene expression was quantified by qPCR in each gonadal, peri-renal, inguinal adipose depot of WT and PRLR^−/−^ non-treated mice (Control) and treated animals (PEG-SMLA, hatched bars) respectively (n = 5–8/group) b, p<0.01. (E) Distribution of adipocyte surfaces in inguinal adipose tissue in untreated WT and PRLR^−/−^ (Control) and treated animals (PEG-SMLA, hatched bars). Statistical analysis was performed. b, p<0.01, c, p<0.001.

We next examined the expression of specific marker genes of adipose tissue. Increase of adipose tissue is reflected by a significant higher leptin expression in gonadal and inguinal adipose tissues after treatment by leptin-antagonist whatever the genotype of animals (Figure 3A).

Then we analyzed key adipose differentiation gene expression to determine the nature of increased adipose tissue mass in PEG-SMLA treated mice. We studied different adipogenic markers, a very early one, the 30-zinc finger transcription factor (*ZFP423*), an early one as Peroxisome Proliferator-Activated Receptor Gamma (*PPARγ*) and a late gene, *aP2*. All markers exhibited the same expression profile in gonadal and peri-renal adipose tissues whatever the genotype with or without PEG-SMLA treatment (Figure 3B, C and D). However, in inguinal adipose tissue, PEG-SMLA injections enhanced adipose differentiation as reflected by the increase of all three adipose markers expression (Figure 3B, C and D).

We subsequently examined inguinal adipose tissue histology and quantified adipocyte surface in adipose depots by histomorphological analysis. Six fields of hematoxylin-eosin stained sections per animal were analyzed at random, adipocyte surface was determined using the Image J software (Figure 3E). Thereafter, adipocytes were classified in different categories according to their surface (from 100 to 8000 μm^2^). In untreated animals of both genotypes, 40% of adipocytes had a surface lower than 500 μm^2^ while they represented only 30% after PEG-SMLA treatment in WT but not in PRLR^−/−^ mice. As expected, adipocytes of WT animals receiving PEG-SMLA treatment were hypertrophic as compared to those of control mice (adipocytes larger than 2000 μm^2^). Statistical analysis on more than 5000 independent determinations shows that inguinal adipose depots from PRLR^−/−^ mice displayed the same proportion of small adipocytes as compared to those of WT mice. Under PEG-SMLA treatment, we observed that PRLR^−/−^ treated mice presented the same distribution of adipocyte size in adipose tissue of both genotypes of non-treated animals. The increase of the adipocyte size appears to be the result of PEG-SMLA treatment only on WT mice, so this treatment failed to modify the adipocyte size distribution in PRLR−/− mice.

### Glucose Intolerance Development after Antagonist Treatment

**Figure 4:**
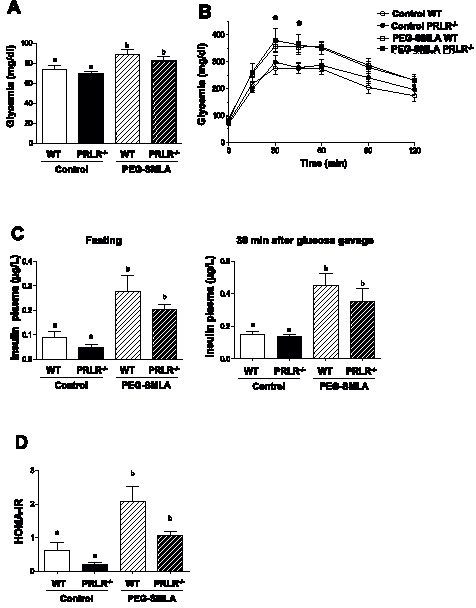
PEG-SMLA treatment induced glucose intolerance and insulin resistance on WT and PRLR−/− animals. (A) Fasting glycemia in saline treated and PEG-SMLA treated mice. b, *p<0.05. (B) Oral glucose tolerance test (2g/kg) was performed in WT and PRLR^−/−^ mice PEG-SMLA treated or not. (C) Fasting (left panel) and 30 minutes after glucose gavage (right panel) plasma insulin levels. (D) HOMA-IR index reflecting insulin resistance is calculated as follows: fasting plasma glucose (mg/dl) × fasting insulin (mU/L)/405.

To determine whether PEG-SMLA treatment affects glucose homeostasis, we performed oral glucose tolerance test (OGTT). Ten-week-old WT and PRLR^−/−^ mice injected with leptin antagonist presented fasted hyperglycemia (Figure 4A) compared to control saline injected mice. This suggests a glucose tolerance impairment confirmed by OGTT shown in Figure 4B. Insulin plasma concentration was determined in a fasted state and 30 minutes after glucose gavage. Mice treated by PEG-SMLA had higher insulin levels at the both 0 and 30 minutes compared to non-treated mice (Figure 4C). Calculated HOMA-IR index is largely amplified in treated animals providing additional support for impaired insulin sensitivity (Figure 4D).

### Metabolic Parameters in mice Treated by Leptin Antagonist

Leptin antagonist injection did not induce change in transaminases (ALAT and ASAT) levels indicating an unaltered hepatic function (Table 1). Lipid serum profile was also studied in order to appreciate the changes in adipose tissues. A slight decrease of the concentrations of non-esterified fatty acids (NEFA) was observed under control conditions only in PRLR^−/−^ mice due to the potential utilization by the muscle or a reduced lipolysis. However, these NEFA concentrations were not modified in PEG-SMLA treated animals of both genotypes (Table 1) suggesting that lipolysis adipose tissue activity did not vary after the treatment. However, PEG-SMLA injection induced a clear increase in circulating triglycerides and cholesterol levels both in WT and PRLR^−/−^ mice according to the mass changes. Altogether, these effects on plasma triglycerides were not associated with liver injury/dysfunction.

**Table 1:** Metabolic parameters in WT and PRLR^−/−^ mice after saline or PEG-SMLA treatment.

	WT control (5)	PRLR^−/−^ control (6)	WT treated (7)	PRLR^−/−^ treated (8)
NEFA (mmol/L)	0.75±0.07^a^	0.52±0.04^b^	0.80±0.04^a^	0.76±0.06^a^
Triglycerides (mmol/L)	1.01±0.17^a^	0.91±0.09^a^	1.63±0.13^b^	1.61±0.18^b^
Cholesterol (mmol/L)	3.17±0.07^a^	3.13±0.13^a^	4.45±0.21^b^	3.83±0.17^c^
ALAT (U/L)	34.60±1.03	32.67±1.38	34.14±2.01	39.86±5.11
ASAT (U/L)	140.40±17.93	171.50±45.80	170.70±25.77	146.0±16.28

Number of mice appears in brackets. Data are represented as mean ± SEM. Different letters within each line indicate statistically different groups.Statistical analyses were performed between the all groups. b, p<0.001; c, p<0.05.

### The Effect of PEG-SMLA Treatment on Trabecular Bone

We have recently shown that PEG-SMLA treatment in both C57BL/6J [16] and in αMUPA, along with their genetic background control (FVB/N) mice [13], revealed a significant increase in trabecular and cortical parameters measured in both the lumbar vertebrae and tibiae, as well as a significant increase in tibia biomechanical parameters [16]. Those findings raised a question: will PRLR^−/−^ mice respond similarly? This effect was tested in the present work and the structural characteristics of trabecular bone from the four groups are presented in Table 2. LV3 and LV4 were analyzed by micro-CT. Both untreated mice groups showed no difference in BV/TV, Tb. Th., Tb. Sp. and Tb. N. However, bone volume fraction and trabecular number were all higher in the PEG-SMLA-treated PRLR^−/−^ mice and WT mice compared to the control groups, whereas trabecular separation was significantly lower in the treated groups. In LV3 analysis, all these results were statistically significant, except for the trabecular separation in PEG-SMLA-treated WT that was lower (not significantly) than the control group. In PRLR^−/−^ treated mice, BV/TV was elevated by 32%. The parameter that mostly contributed to these results was trabecular number, which was higher by 26%, while trabecular thickness was higher by only 5%. Similar results were obtained in the WT PEG-SMLA-treated mice, BV/TV was elevated by 43%, trabecular number was higher by 32%, while trabecular thickness was higher by 8.6%. LV4 analysis yielded similar results.

**Table 2:** Trabecular bone parameters of third and fourth lumbar vertebrae (LV3 and LV4) of PEG-SMLA *vs* saline-injected PRLR^−/−^ and WT mice.

LV3	WT control	PRLR^−/−^ control	WT treated	PRLR^−/−^ treated
BV/TV (%)	18.56±0.46^a^	18.56±0.650^a^	26.56±1.060^b^	24.5±0.930^b^
Tb. Th. (μm)	0.069±0.001^c^	0.071±0.001^bc^	0.075±0.001^a^	0.075±0.001^ab^
Tb. Sp. (μm)	0.247±0.006^a^	0.273±0.010^b^	0.231±0.005^a^	0.243±0.005^a^
Tb. N (1/μm)	2.65±0.070^a^	2.58±0.080^a^	3.49±0.110^b^	3.26±0.090^b^
**LV 4**				
BV/TV (%)	19.54±0.25^a^	20.5±1.6^a^	26.43±1.16^b^	26.04±1.03^b^
Tb. Th. (μm)	0.072±0.0009^a^	0.073±0.001^a^	0.075±0.001^a^	0.076±0.0008^a^
Tb. Sp. (μm)	0.256±0.009^ab^	0.272±0.01^a^	0.226±0.006^b^	0.234±0.007^b^
Tb. N (1/μm)	2.71±0.05^a^	2.75±0.14^a^	3.51±0.12^b^	3.4±0.11^b^

Trabecular bone volume (BV)/tissue volume (TV) (%), trabecular number (Tb.N) (1/μm), trabecular thickness (Tb-Th) (μm), and trabecular separation (Tb.Sp) (μm). Data are mean ± SEM. Results shown in each row that are not designed with the same letter are statistically different, p*<*0.05, n = 6.

## Discussion

Beside the many functions ascribed to PRL, its involvement has been best characterized with reproduction, but it is now established that PRL also exerts metabolic actions [17]. We demonstrated earlier that despite increased food intake in both chow diet and high fat diet (HFD), PRLR^−/−^ mice remained leaner than controls and were protected against HFD-induced obesity with a marked reduction in adiposity. The relative resistance to HFD-induced obesity was accompanied by a more favorable carbohydrate homeostatic profile in PRLR^−/−^ mice, consistent with the major implication of PRL signaling in energy balance [11]. These mice displayed reduced fat mass associated with appearance of massive brown-like adipocyte foci in perirenal and subcutaneous fat depots under HFD. These effects were essentially peripheral due to enhanced energy expenditure and increased metabolic rate, then we planned to decipher the central PRL effect. Thus, we took advantage of the availability of a leptin superactive antagonist to examine its consequence on PRLR^−/−^ mice.

In this study, the PEG-SMLA treatment induced a robust and transient weight gain driven by an increase in food intake in both genotypes. Due to the pegylation process of this superactive compound its *in vivo* activity was elevated and persistent as already described [12]. It is noteworthy that during the first week of treatment the body weight is dramatically increased as far as the food intake, whereas during the second week of treatment these effects are less pronounced, as already described in transgenic murine urokinase-type plasminogen activator mouse model [13]. The genetic background of the PRLR^+/+^ and PRLR^−/−^ mice is also another important parameter to be considered, since it has been described that the response to diet is background dependent [18]. However, even on a 129Sv inbred mouse strain our results demonstrate that these PRLR^−/−^ mice respond to the PEG-SMLA at a lesser extend to other mouse models such as C57BL/6 (30% *vs* 40%) [12]. Along this line, the different fat depots are also increased, these results are in accordance with those published elsewhere [13]. However, the early and late differentiation markers of adipose tissue are only enhanced in the inguinal fat pad in WT mice as well as in PRLR^−/−^ mice. This demonstrates that this depot is the first adipose tissue highly adapted to store excess energy in the form of triglycerides whatever the presence of PRL signaling. In addition PEG-SMLA injected WT mice had a greater number of large adipocytes than in untreated WT and PRLR^−/−^ mice. The distribution of large adipocytes was reflected by a nearly 2.5-fold increase in mean surface area of adipocytes of treated WT mice as compared to untreated animals leading to an adipocyte hypertrophy. However, as no statistical difference appeared in PRLR^−/−^ PEG-SMLA treated animals this phenotype combined to the increased expression of all tested adipose markers suggests adipocyte hyperplasia.

In both WT and PRLR^−/−^ mice, glucose intolerance and an impairment of insulin sensitivity were observed demonstrating that the absence of PRLR does not impact the weight gain, the food intake, the adipocyte fate/differentiation and metabolic parameters. These results do not fit with previous data showing a small, but progressive reduction in the rate of weight gain and a reduction in abdominal fat mass in PRLR^−/−^ mice on chow diet [15]. However, these observations have been documented with 16 week-old animals, whereas our present study was performed on 8 week-old mice.

Bone analysis revealed that PRLR^−/−^ mice exhibit the same values of trabecular BV/TV as their parental strain 129/SvJ. Syberg et al [19] showed that 129/SvJ mice have mechanically tested stronger femur, higher bone mineral density, and higher trabecular BV/TV compared to C57BL/6J mice. PEG-SMLA treatment elevated significantly trabecular BV/TV of both PRLR ^−/−^ mice and WT compared to the untreated animals suggesting that the trabecular bone of the treated animals is denser (i.e. has a higher material content per volume of tissue), and is therefore likely to be more effective in resisting the loads acting on the vertebrae. The positive effect of leptin inhibition on trabecular bone fraction and weight gain in diet-induced obesity resistant PRLR^−/−^ mice, suggests that leptin and PRL signaling pathways are acting separately from each other.

Cessation of PEG-SMLA treatment on day 21, affected weight of WT and PRLR^−/−^ mice which returned to almost control level and both serum insulin and OGTT also returned to normal levels. These results indicate that PEG-SMLA enabled reversible induction of a state of leptin deficiency in PRLR^−/−^ mice as well as in other mouse strains previously shown [12], [13], [20].

Altogether, our results demonstrated that PRLR^−/−^ mice respond dramatically to leptin antagonist, suggesting that the resistance to HFD shown previously [11] is not linked to leptin action.
